# Bioinformatics analysis reveals the landscape of immune cell infiltration and novel immune-related biomarkers in moyamoya disease

**DOI:** 10.3389/fgene.2023.1101612

**Published:** 2023-05-17

**Authors:** Lei Cao, Yunzheng Ai, Yang Dong, Dongpeng Li, Hao Wang, Kaiwen Sun, Chenchao Wang, Manxia Zhang, Dongming Yan, Hongwei Li, Guobiao Liang, Bo Yang

**Affiliations:** ^1^ Department of Neurosurgery, The First Affiliated Hospital of Zhengzhou University, Zhengzhou, China; ^2^ Department of Neurosurgery, General Hospital of Northern Theater Command, Shenyang, China

**Keywords:** MMD, immune infiltration, biomarkers, bioinformatics analysis, WGCNA

## Abstract

**Objective:** This study aimed to identify immune infiltration characteristics and new immunological diagnostic biomarkers in the cerebrovascular tissue of moyamoya disease (MMD) using bioinformatics analysis.

**Methods:** GSE189993 and GSE141022 were downloaded from the GEO database. Differentially expressed gene and PPI analysis were performed. After performing WGCNA, the most significant module associated with MMD was obtained. Next, functional pathways according to GSEA, GO, and KEGG were enriched for the aforementioned core genes obtained from PPI and WGCNA. Additionally, immune infiltration, using the CIBERSORT deconvolution algorithm, immune-related biomarkers, and the relationship between these genes, was further explored. Finally, diagnostic accuracy was verified with ROC curves in the validation dataset GSE157628.

**Results:** A total of 348 DEGs were screened, including 89 downregulated and 259 upregulated genes. The thistle^l^ module was detected as the most significant module associated with MMD. Functional analysis of the core genes was chiefly involved in the immune response, immune system process, protein tyrosine kinase activity, secretory granule, and so on. Among 13 immune-related overlapping genes, 4 genes (*BTK, FGR, PTPN11,* and *SYK*) were identified as potential diagnostic biomarkers, where *PTPN11* showed the highest specificity and sensitivity. Meanwhile, a higher proportion of eosinophils, not T cells or B cells, was demonstrated in the specific immune infiltration landscape of MMD.

**Conclusion:** Immune activities and immune cells were actively involved in the progression of MMD. *BTK, FGR, PTPN11*, and *SYK* were identified as potential immune diagnostic biomarkers. These immune-related genes and cells may provide novel insights for immunotherapy in the future.

## Introduction

Moyamoya disease (MMD) shows stenosis or obstruction at the terminal portion of the internal carotid artery in angiography and also an abnormal vessel network formed at the base of the brain (2012). Epidemiologically, this rare and chronic cerebral vascular disease has a higher prevalence predominantly in Japan and East Asia (Kim, 2016). Patients chiefly suffering from MMD include children (from 5 years to 9 years) and young adults (from 35 to 45 years) (Kim, 2016). However, the pathogenesis of cerebral vascular disease still remains unclear (Bang et al., 2016; Fox et al., 2021). Microarray technology was used to detect genetic alterations, and bioinformatics analysis tools were used to explore the potential etiology and pathogenesis at the genome level. It is an effective method in large-scale research of gene expression. There is increasing evidence that genetic factors have a crucial role in the progression of MMD (Wang X. et al., 2020), and previous studies have shown that the disease is commonly associated with neurofibromatosis type I (Koc et al., 2008), Down syndrome (Fukuyama et al., 1992), and other inherited diseases. Additionally, *RNF213* has been identified as the first susceptible gene for MMD using a genome-wide linkage and exome analyses by two independent studies (Kamada et al., 2011; Liu et al., 2011). Moreover, transcriptomic study (Xu et al., 2022) and the lncRNA–mRNA co-expression pattern (Zhao et al., 2022) have been investigated to provide new insights into the pathogenesis of MMD. Hence, gene expression profile analysis could be used to better unveil the genetic and molecular mechanisms associated with MMD development. Immune cell infiltration provides a new perspective for exploring the immune mechanism of diseases, and several non-tumor studies have revealed the unique role in the occurrence, development, and prognosis of diseases (Deng et al., 2020; Liu et al., 2020; Maekawa et al., 2021; Hou et al., 2022). However, due to the low incidence rate of diseases and the difficulty in obtaining samples, only few previous research studies have analyzed the genetic alterations, the landscape of overall immune infiltration, and the relationship of hub genes and immune cells in microarray data.

**FIGURE 1:**
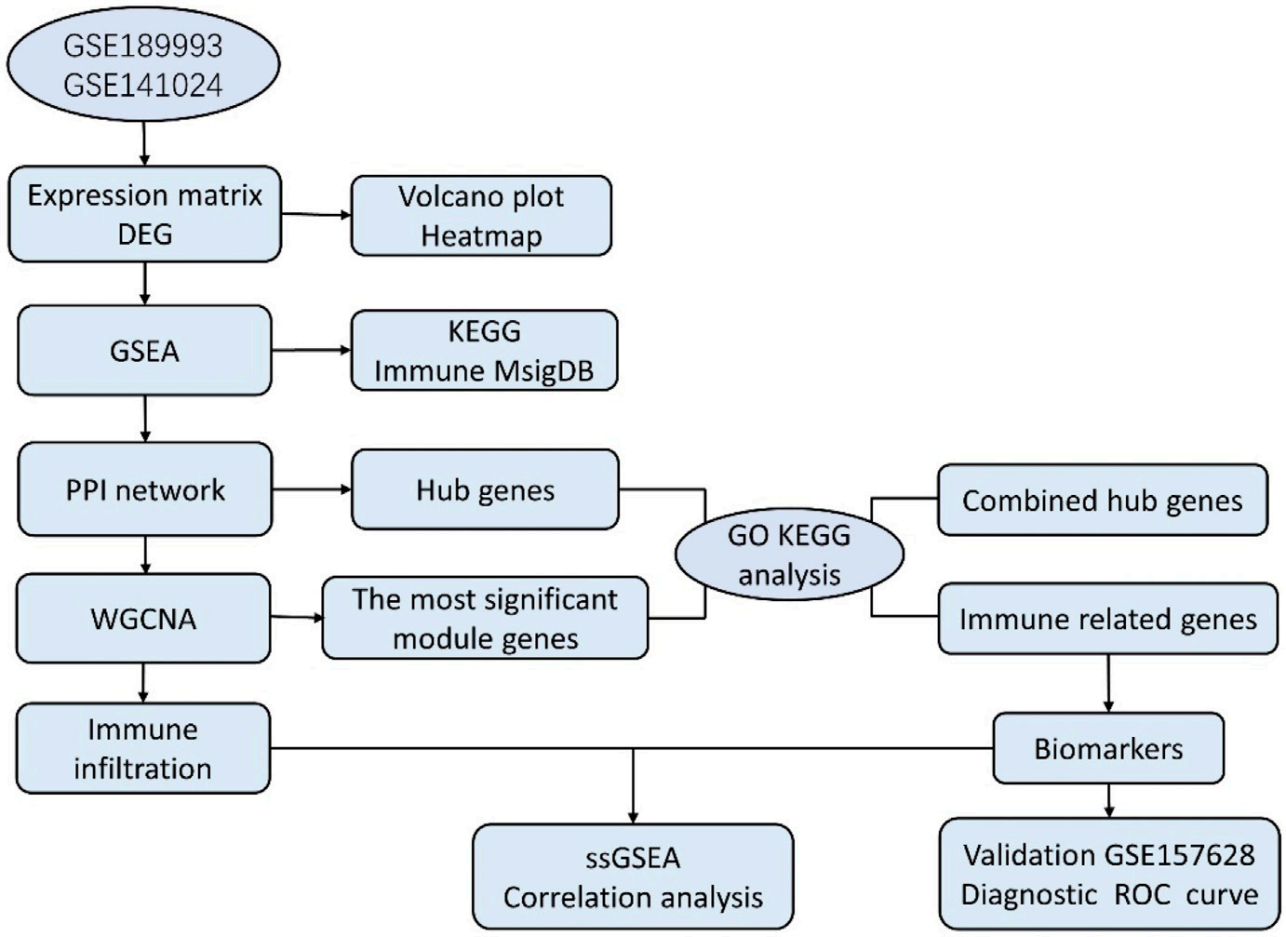
Workflows of bioinformatics study.

In our study, the microarray datasets GSE189993 and GSE141022 were downloaded and combined. After analyzing the data between MMD and the control group, we obtained the differentially expressed genes (DEGs), and another GSE157628 was downloaded and utilized as an external validation dataset. The current work conducted WGCNA based on MMD analysis first, and we explored the 22 infiltrating immune cell components for the first time using the combined dataset of Gene Expression Omnibus (GEO). The project aims to explore immune infiltration characteristics and identify new immunological diagnostic biomarkers. This study sheds new light on the pathogenesis and diagnosis of MMD. Bioinformatics analysis workflows of the study are shown as follows (Figure 1).

## Materials and methods

### Data collection and preprocessing

We searched and selected two microarray datasets in the GEO database. GSE189993 and GSE141022 were downloaded, and the gene expression data of the middle cerebral artery were analyzed. GSE189993 consists of 21 MMD and 11 non-MMD patients, and GSE141022 consists of four MMD and four non-MMD patients. After normalization, we used the SVA package in R script for background correction, and the ComBat function was used to remove the batch effects. After correction, the distribution of the data was visualized by principal component analysis (PCA), and PCA illustrated that different batches were at the same level. We used the mean as the unique expression value for duplicate gene symbols and the limma package to identify DEGs. *p* < 0.05 was considered significant. |log2 FC (fold change) | >1 was the criterion for identifying DEGs.

### Gene set enrichment analyses (GSEA) of the expression matrix

GSEA is a bioinformatics tool used to analyze enriched gene sets, KEGG pathways, and immunological signatures, as well as for analyzing genome-wide expression data. Genes in the gene set share the same function and other features. GSEA could screen the functional enrichment score for all expression profiles rather than different expression genes alone. A good score was presented in terms of rank-based and p-value-based pathway enrichment, and no false prediction was found (Nguyen et al., 2019). GSEA software was obtained from the official website (Subramanian et al., 2005). All gene expression profiles were divided into two groups, and the c2.cp.kegg.v7.4.symbols.gmt subset obtained from MsigDB (Liberzon et al., 2011) was used to evaluate molecular mechanisms. The minimum and maximum number of gene sets ranged from 5 to 5000, and 1000 was set as the threshold of permutations. *p* < 0.05 and FDR<0.25 were considered significant. To further explore the immune pathways, the c7.immunesigdb.v7.4.symbols.gmt subset was also obtained from MsigDB to evaluate immune-related pathways. The minimum and maximum number of gene sets ranged from 5 to 5000, and 1000 was set as the threshold of permutations. *p* < 0.01 and FDR<0.1 were considered significant.

### Protein–protein interaction (PPI) network and hub gene analysis

We used the STRING online database to construct the PPI network (von Mering et al., 2003). The network was drawn by Cytoscape based on the results of the STRING analysis, and the most important module was identified by MCODE plugin (Bader and Hogue, 2003), and hub genes were identified by the hub gene plugin. The top 50 genes were selected as hub genes from five algorithms, namely, Closeness, Degree, MCC, MNC, and EPC. The real core genes were obtained from the intersection of the result. The default parameters for MCODE were selected as the criterion.

### WGCNA

WGCNA is usually used to analyze the gene expression patterns. According to the WGCNA algorithm, we performed Pearson’s correlation and average linkage first. β was known as the soft-thresholding parameter, and it emphasized strong correlations between genes and penalized weak correlations. Next, we transformed the adjacency matrix into a topological overlap matrix (TOM) and calculated the corresponding dissimilarity (1-TOM) after confirming the soft-thresholding power. A gene’s network connectivity could be measured by TOM. To classify the genes sharing similarity into gene modules, we then carried out average linkage hierarchical clustering in accordance with TOM-based dissimilarity, and gene dendrograms should have had a minimum size of 30. To further explore these modules, the dissimilarity of the module eigengenes was computed, and we merged some modules in the module dendrogram after choosing a cut line. Last, the relationship between specific clinical features and the modules was analyzed.

### Function analysis of core genes

Gene Ontology (GO) and KEGG are the most important bioinformatics tools for the analysis of gene annotation and biological processes. First, the identified PPI hub genes and WGCNA gene set were merged together to obtain the combined core genes. Based on these core genes, GO terms and KEGG pathway enrichment were performed, respectively. Next, we obtained immune gene symbol lists from the website. In order to identify immune DEGs, ImmPort (The Immunology Database and Analysis Portal database) was selected to identify immune-related genes. The ImmPort gene set contains 2,489 immune-related genes. The overlapping genes were obtained from the intersection of three datasets, hub genes of DEGs, and hub genes of the most important molecular and immune-related gene set. GO terms and KEGG pathways were analyzed as well based on these immune-related core genes. *p* < 0.05 was considered significant for GO terms and KEGG pathway analysis.

### Evaluation of immune-related biomarkers and ROC curves

Finally, another dataset (GSE157628) was utilized to validate immune-related core genes.

Expression values from the original combined expression matrix in samples of GSE189993 and GSE141022 were extracted. Expression values of the same genes from microarray data in samples of GSE157628 were extracted as well. The expression pattern was compared between the two groups. To evaluate diagnostic accuracy, the receiver operating characteristic (ROC) curve was applied, and we calculated the area under the curve (AUC). Ideally, the AUC should be 1, and values over 0.5 are considered predictive.

### CIBERSORT analysis and correlation with immune biomarkers

The cell-type identification by estimating relative subsets of the RNA transcript algorithm, also called CIBERSORT (Newman et al., 2015), is widely used in evaluating cell fractions. Based on our gene expression profile, we selected the computational method to calculate the score of immune infiltrating cells. The fractions of 22 types of infiltrating immune cells in each sample were visualized by a bar graph, and the correlation between immune cell subtypes and gene expression was visualized by a correlation heat map.

To further confirm immune-related genes as potential diagnostic immune biomarkers, we divided the 60 samples into the MMD and non-MMD groups; the MMD group had 35 patients and the non-MMD group had 25 patients. The immune scores of 22 infiltrating immune cell subtypes were compared. After analyzing immune infiltration, we performed Spearman’s correlation to determine the relationship between the expression levels of identified immune biomarkers and 22 subtypes of infiltrating immune cells.

### Statistical analysis

Data analysis and statistical analysis were conducted by R Studio (version 4.1.2). R package “ggplot2” and “pheatmap” and other packages were used to generate the pictures of volcano plots, heat maps, and principal component analysis. Box plots were drawn by GraphPad Prism 7.0 software using the Mann–Whitney *U* test. The ROC curve was performed with SPSS 17.0 software. The results were imported into Sangerbox (Shen et al., 2022), and the data were visualized. In the statistical analysis, we selected False Discovery Rate (FDR) for multiple corrections, and *p* < 0.05 was considered statistically significant.

## Results

### Data preprocessing and DEG analysis

**FIGURE 2:**
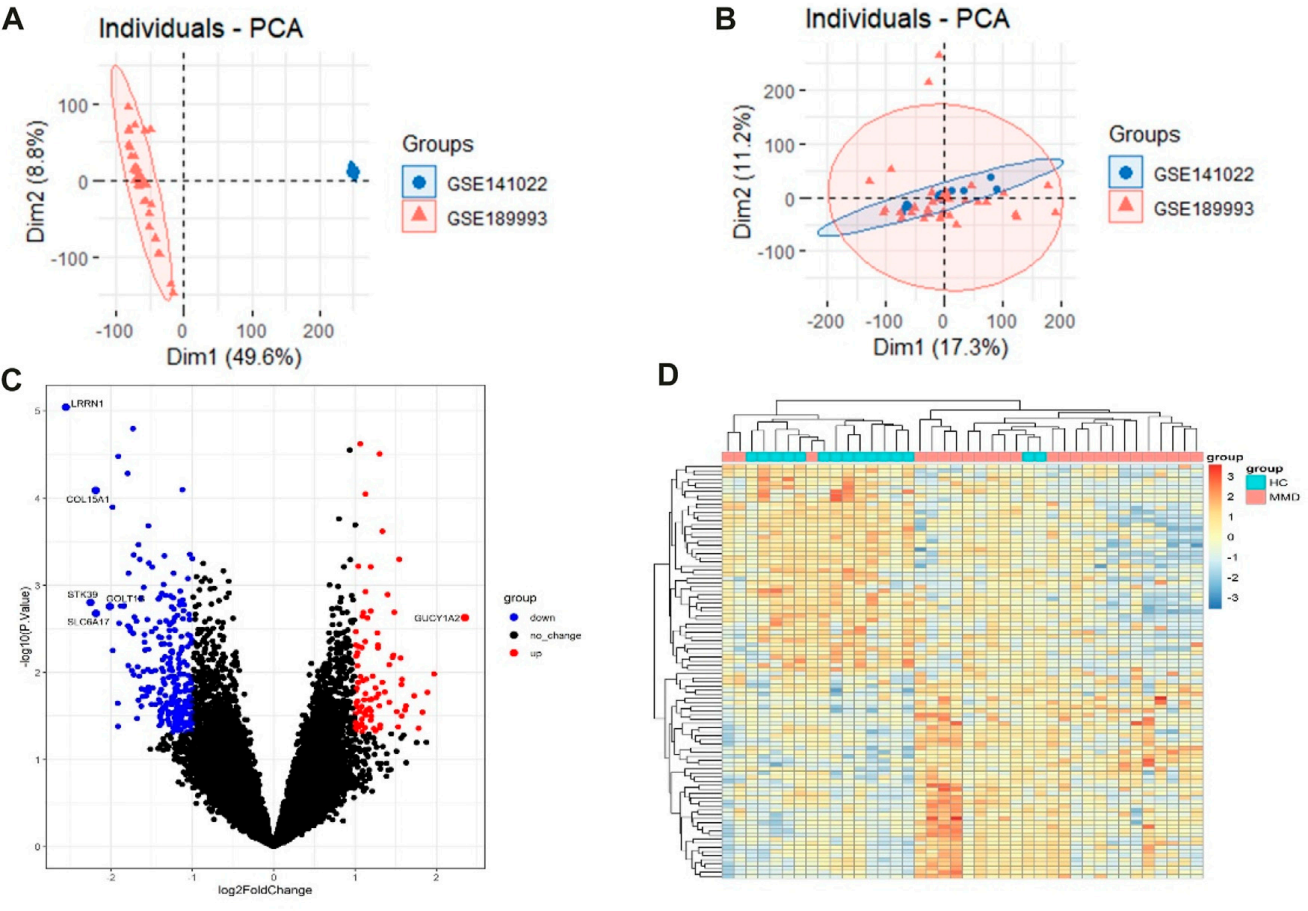
**(A)** Pre-processing PCA of two selected datasets. **(B)** Post-processing PCA of two selected datasets. **(C)** Volcano plot depicting the DEGs between two groups, genes significantly upregulated in the MMD group are indicated by red dots, genes significantly downregulated in the MMD group are indicated by blue dots, and black dots indicate non-DEGs. **(D)** Heat map of DEGs; each column represents a sample, and each row represents a DEG.

The raw data were processed, and the results showed that the samples from each dataset were comparable (Figures 2A, B). After normalization, we found 348 DEGs between MMD and control groups, which consisted of 89 upregulated genes and 259 downregulated genes. The analysis results of the expression matrix are depicted in the volcano plot (Figure 2C). The top 50 and last 50 DEG genes are depicted in the heat map (Figure 2D) (Supplementary Table S1).

### Gene Set Enrichment Analysis

**FIGURE 3:**
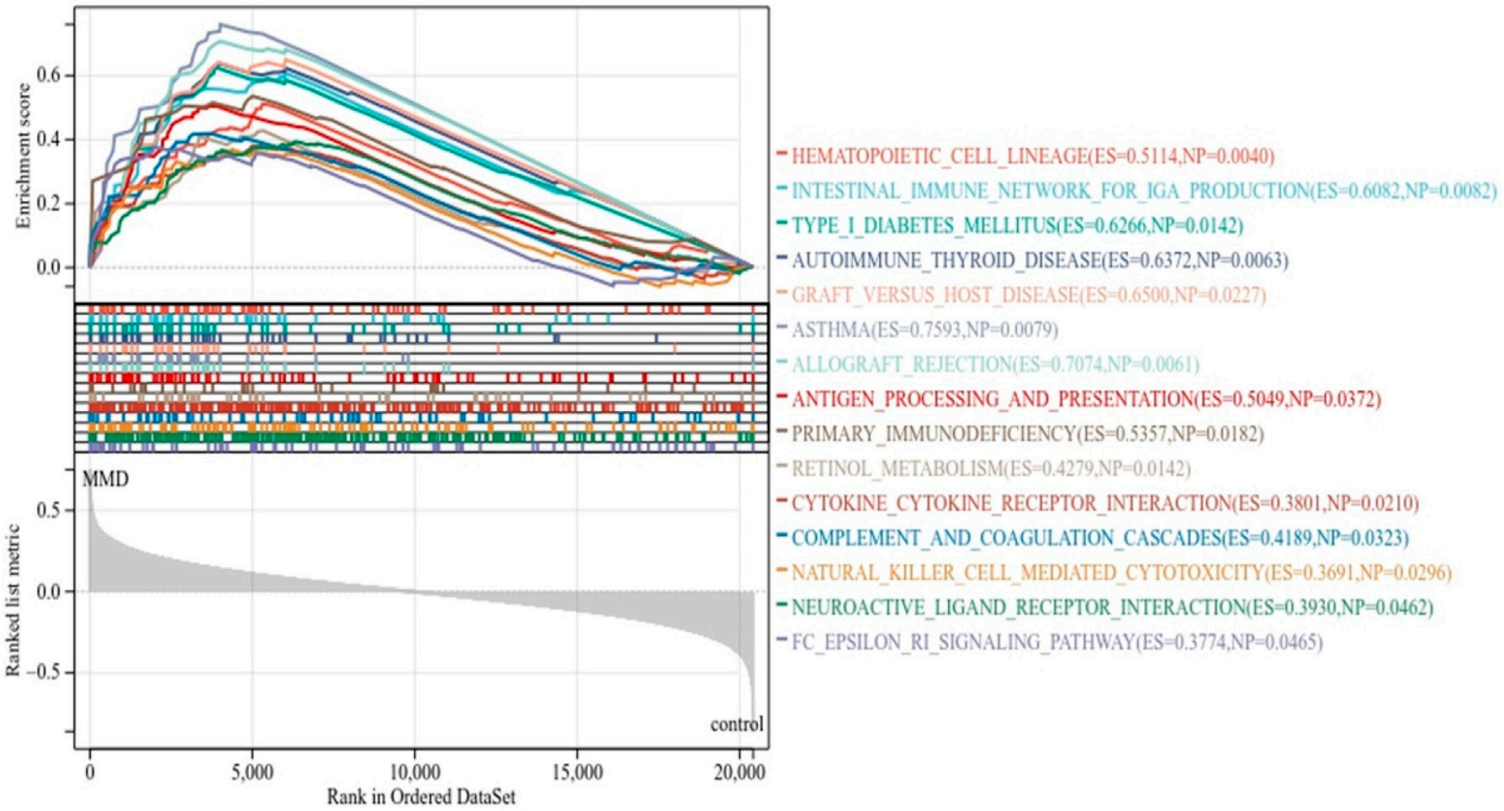
KEGG enrichment analysis by GSEA. The line means enrichment profile. Peak 6 appears at the front of the sequenced gene set (ES > 0) indicating that the pathway is upregulated, and when it appears at the back (ES < 0), it indicates that the pathway is downregulated. The vertical line marks the position of each member in the gene set in the gene sequencing list. The red part represents overexpressed genes in the experimental group, whereas the blue part represents overexpressed genes in the control group. The gray area diagram shows the distribution of all gene rank values after sequencing.

As expected, the exported results of GSEA-based enrichment analysis illustrated a strong correlation between immune cells and immune activities. According to the KEGG enrichment analysis, there were 15 enriched terms, and they were all upregulated (*p* < 0.05 and FDR<0.25), mainly involving primary immune-related pathways, including natural killer cell-mediated cytotoxicity, antigen processing and presentation, primary immunodeficiency, and the Fc epsilon RI signaling pathway; pathways related to immune-associated diseases include autoimmune thyroid disease, type-I diabetes mellitus, graft *versus* host disease, asthma, and allograft rejection; pathways related to cytokine activation include cytokine–cytokine receptor interaction and neuroactive ligand receptor interaction. The immune-related pathways results are visualized in Figure 3 (Supplementary Table S2).

**FIGURE 4:**
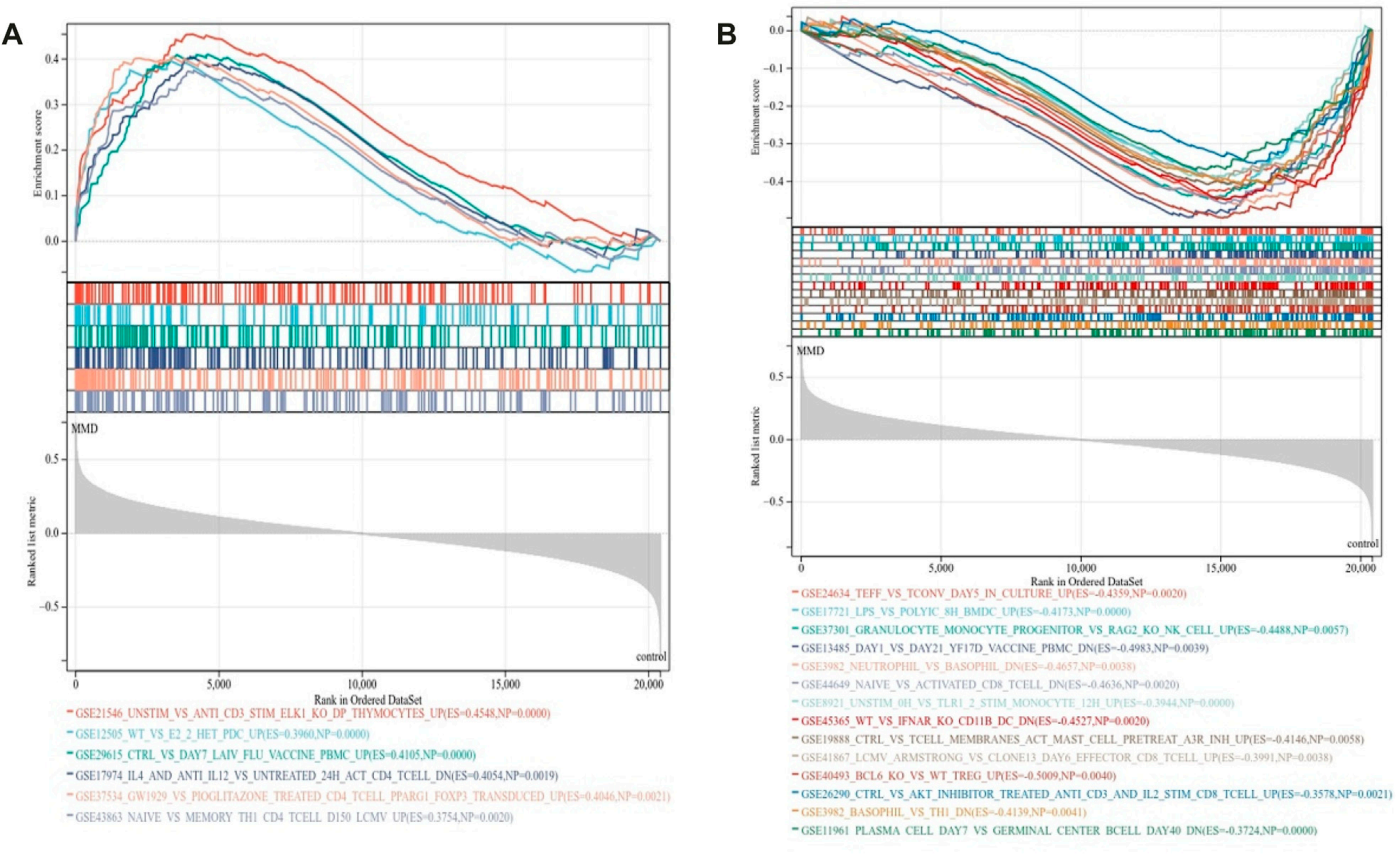
Immunological signature enrichment analysis by GSEA. **(A)** Enriched immunological signature gene sets in the activated pathway. **(B)** Enriched immunological signature gene sets in the suppressed pathways.

To further characterize the signature of immune activities related to the pathology of MMD, GSEA was used again to identify the immunological pathways enriched in immune signature gene sets. The criteria *p* < 0.01 and FDR <0.15 showed that 20 prominent immune pathways were enriched totally. There were 6 activated pathways (Figure 4A) and 14 suppressed pathways (Figure 4B) among them. The gene sets in CD4 T cell, CD8 T cell, Th1 cells, monocytes, mast cells, dendritic cells (DCs), and peripheral blood mononuclear cells were mainly enriched (Supplementary Table 3).

### Protein–protein interaction analysis

**FIGURE 5:**
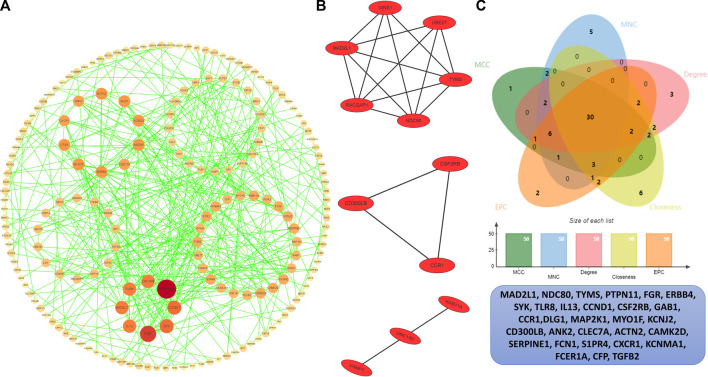
PPI analysis. **(A)** PPI network of the interactions, where each dot represents each DEG, the color represents the degree of each DEGs, the redder the larger degree, and the yellower the smaller degree. **(B)** Top three most significant modules. **(C)** Venn plot of different cytoHubba algorithms and the identified thirty hub genes.

In order to identify the hub genes, 384 DEGs were analyzed with the PPI network (Figure 5A). The three most significant modules were clustered via MCODE (Figure 5B), module 1 was made up of six DEGs, and module 2 and module 3 consisted of three DEGs. According to five commonly used classification algorithms in cytoHubba, the top 50 genes were identified by each algorithm. Finally, we took the intersection of Closeness (top 50), Degree (top 50), MCC (top 50), MNC (top 50), and EPC (top 50) to acquire 30 hub genes (Figure 5C) (Supplementary Table S4).

### Key module of WGCNA

**FIGURE 6:**
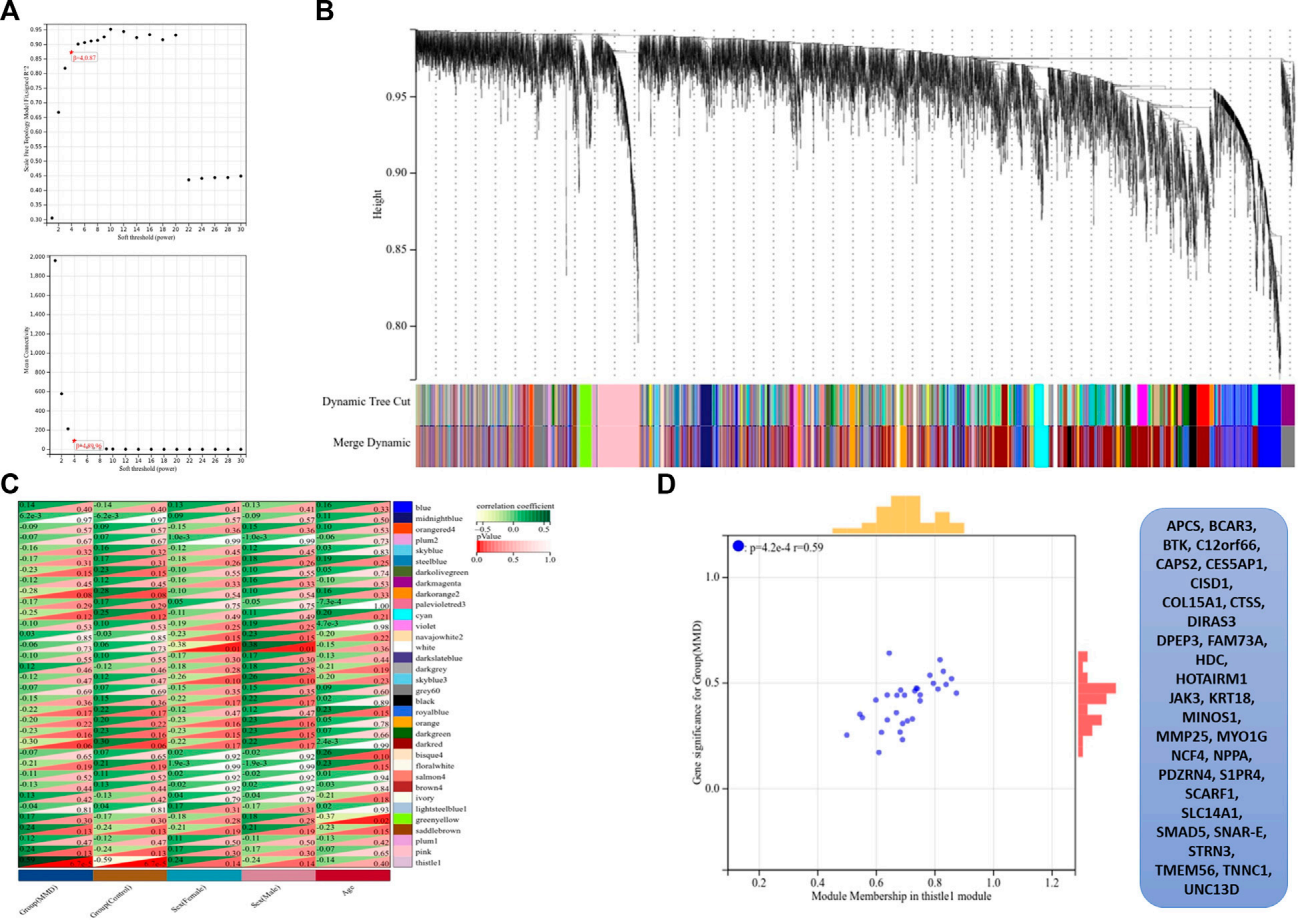
Results of WGCNA. **(A)** Determination of soft-thresholding power, the scale-free index was analyzed, and the mean connectivity was depicted for a variety of soft-threshold powers. **(B)** Hierarchical clustering tree and co-expression modules are depicted according to the measurement of dissimilarity (1-TOM). Each module was coded with different colors, and a group of high related genes were contained in each color-coded module. **(C)** Identification of modules highly related to the clinical traits, and heat map results showed the correlation between clinical characteristics and co-expression modules. **(D)** Selected thistle module and its genes.

The co-expression modules were generated after WGCNA. Gene sets contained in co-expression modules frequently shared high topological overlap similarity. To guarantee a scale-free network, we chose the soft-threshold β = 4 (scale-free *R*2 = 0.87) in the present study. The adjacency matrix was converted into a TOM matrix (Figure 6A), which showed the similarity between different nodes because the weighted correlation was considered in the matrix. Ultimately, we obtained 34 co-expression modules by WGCNA (Figure 6B). In order to select the most important clinical module, we performed correlation analysis between the identified modules and clinical traits (Figure 6C). Among these modules, the thistle^l^ module was selected, and it showed a strong correlation with the MMD group (cor = 0.59 and *p* = 4.2 e–4). Automatically, all the 31 genes contained in this module were identified as the hub genes (Figure 6D) (Supplementary Table S5).

### Functional annotation analysis of hub genes

**FIGURE 7:**
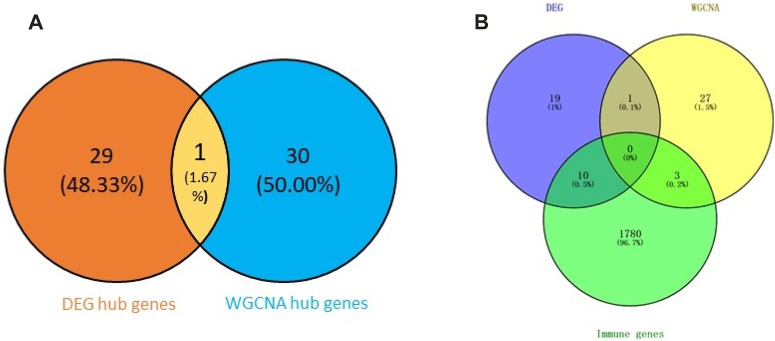
Venn plot of the hub genes. **(A)** Combination of two hub gene sets. **(B)** Overlapping of three gene sets.

**FIGURE 8:**
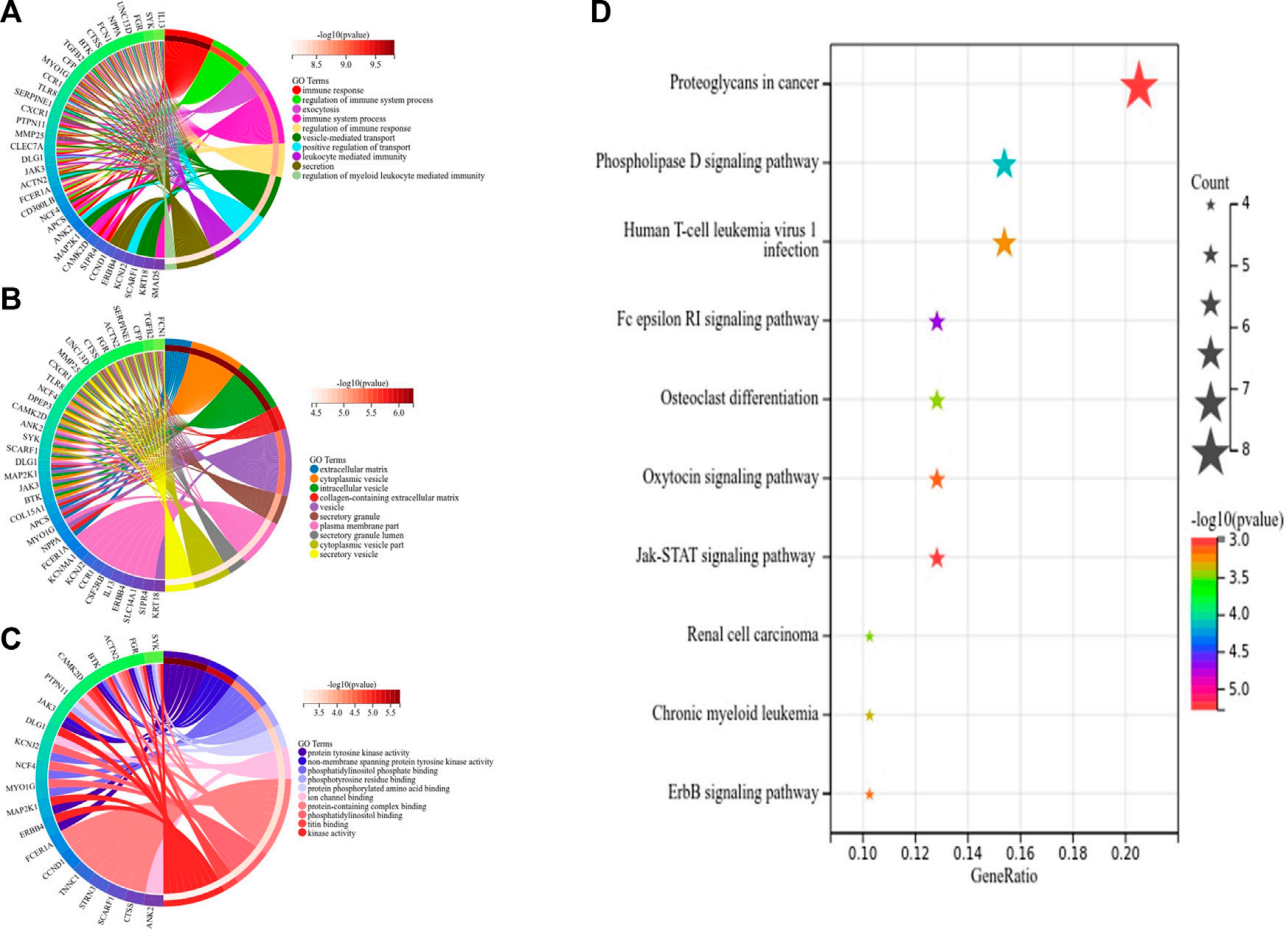
Top 10 GO and KEGG enrichment of core genes. **(A)** Circos plot of BP. **(B)** Circos plot of CC. **(C)** Circos plot of MF. **(D)** Bubble plots of KEGG pathways, and gene counts enriched in the signaling pathway are represented by dot size. The color indicates the significance of *p*-value.

First, we obtained 61 core genes with the combination of the aforementioned two hub gene sets (Figure 7A) (Supplementary Table S6). In order to study the biological process of the core genes, GO and KEGG functional analyses were conducted. The biological process analysis of the hub genes was mainly related to the immune response, immune system process, regulation of the immune response and immune system process, vesicle-mediated transport, exocytosis, secretion, and so on (Figure 8A). Cell component analysis of the hub genes was primarily enriched in the extracellular matrix, cytoplasmic vesicle, intracellular vesicle, secretory vesicle, secretory granule, plasma membrane part, and so on (Figure 8B). Molecular function analysis was primarily enriched in the protein tyrosine kinase activity, phosphatidylinositol phosphate binding, phosphatidylinositol binding, phosphorylated amino acid binding, ion channel binding, titin binding, and so on (Figure 8C). KEGG analysis revealed that the proteoglycans in cancer, human T-cell leukemia virus I infection, phospholipase D signaling pathway, Jak-STAT signaling pathway, Fc epsilon RI signaling pathway, and ErbB signaling pathway were mainly enriched (Figure 8D). The results of the functional analysis revealed that immune activities may be mainly involved in the progression of MMD. The detailed results are illustrated in Figure 8.

**FIGURE 9:**
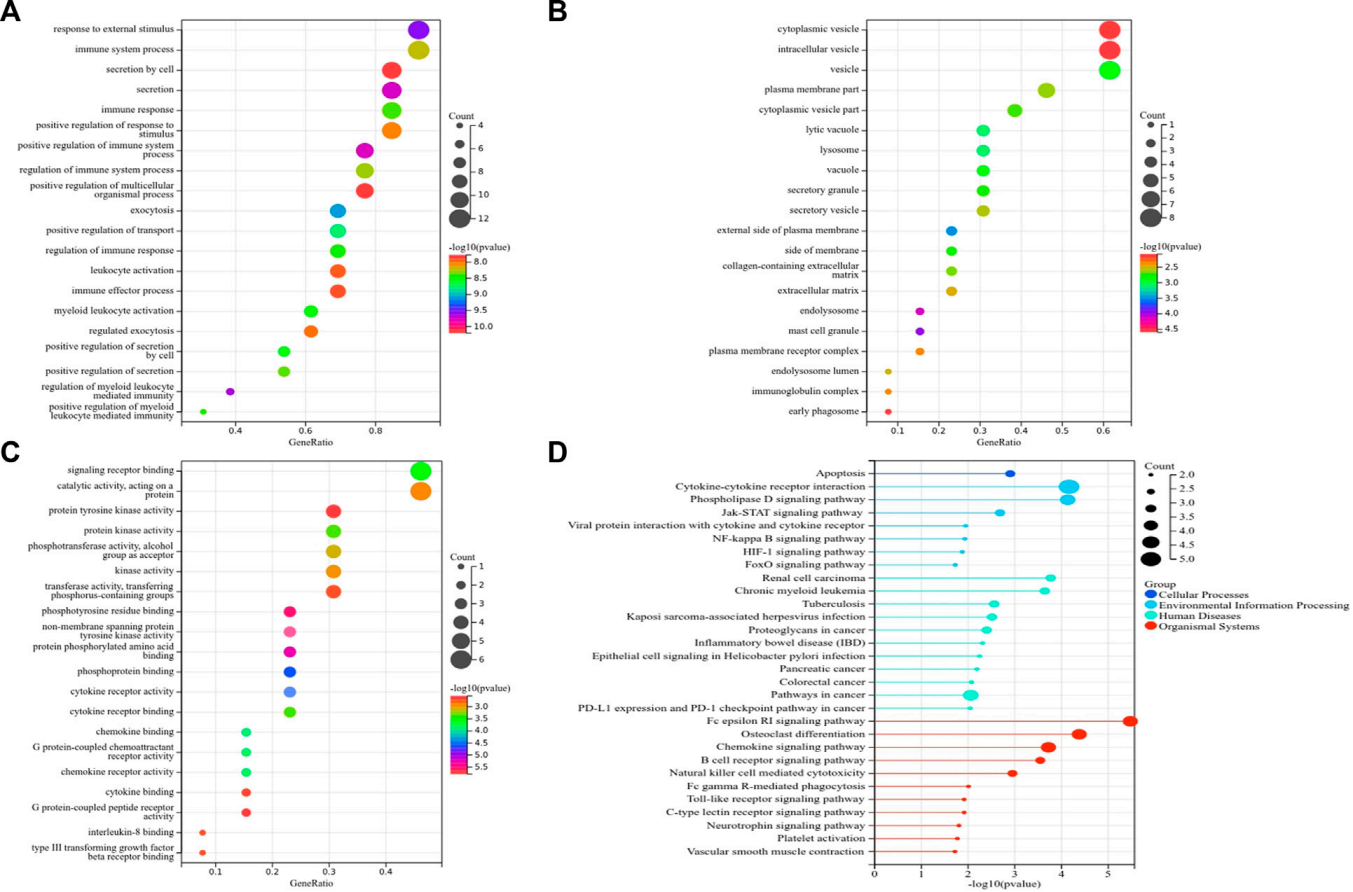
Top 10 GO and KEGG enrichment of immune-related genes. **(A)** Bubble plots of BP. **(B)** Bubble plots of CC. **(C)** Bubble plots of MF. **(D)** Lollipop chart of KEGG pathways. The vertical axis shows the description of GO and KEGG enrichment, and the horizontal axis of GO and KEGG shows the gene ratio and −log10 (*p*-value), respectively. The color intensity indicates the significance of *p*-value, and the circular area indicates gene counts.

Subsequently, we identified 13 immune-related genes overlapped in the PPI hub genes, WGCNA hub genes, and the immune genes set, as shown in the Venn plot (Figure 7B), including *CTSS, NPPA, PTPN11, FGR, SYK, TLR8, IL13, CSF2RB, CCR1, MAP2K1, CXCR1,* and *TGFB2*. To screen biological differences of the immune-related genes between MMD and the control group, GO and KEGG functional analyses for the identified immune genes were performed as well and showed results similar to those of the core genes. GO analysis showed that these immune-related hub genes mainly involved the immune response and immune system process and their regulation, vesicle-mediated transport, exocytosis, secretion in BP (Figure 9A), extracellular matrix, cytoplasmic vesicle, intracellular vesicle, lysosome, mast cell granule and immunoglobulin complex in CC (Figure 9B), protein tyrosine kinase activity, signaling receptor binding, cytokine receptor activity/binding, type-III transforming growth factor beta receptor binding, and G protein-coupled peptide receptor activity in MF (Figure 9C). KEGG analysis revealed that these immune hub genes primarily involved apoptosis, cytokine–cytokine receptor interaction, phospholipase D signaling pathway, Jak-STAT signaling pathway, NF-kappa B signaling pathway, HIF-I signaling pathway, Fc epsilon RI signaling pathway, chemokine signaling pathway, B-cell receptor signaling pathway, NK cell-mediated cytotoxicity, platelet activation, and vascular smooth muscle contraction (Figure 9D). The consistent results revealed a close relationship between MMD and the immune mechanism. The detailed results are illustrated in Figure 9.

### Verification and ROC of immune markers

**FIGURE 10:**
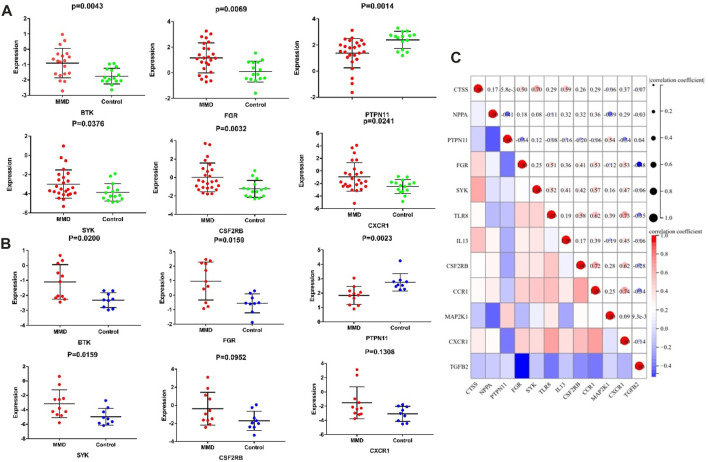
Significantly expressed hub genes. **(A)** Expression values of BTK, FGR, PTPN11, and SYK were extracted from the original expression matrix in samples of GSE189993 and GSE141022. **(B)** Expression values of the same genes were extracted from microarray data in samples of GSE157628. **(C)** Correlation matrix of the expression of 13 immune-related genes.

**FIGURE 11:**
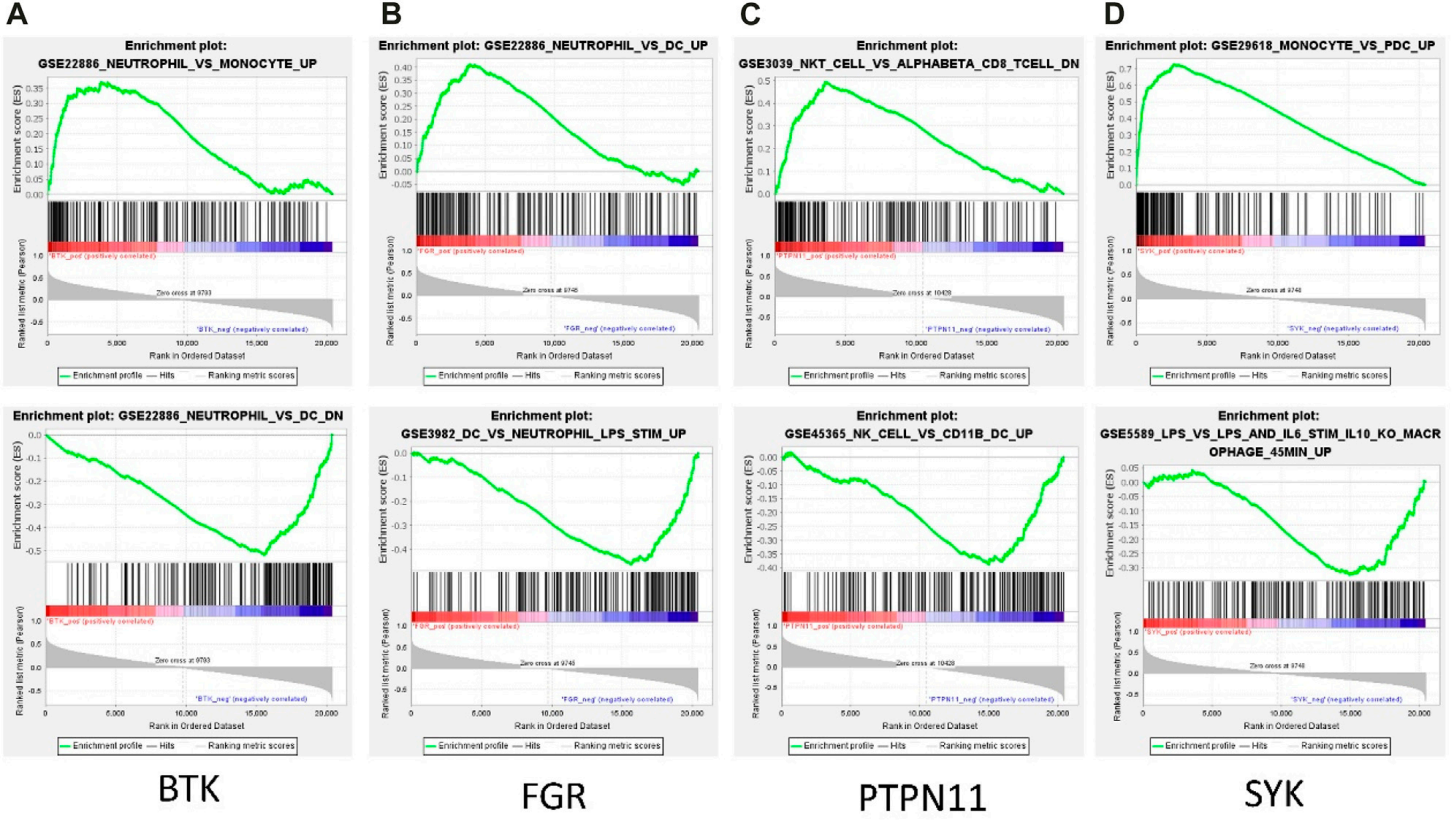
Representative GSEA enrichment score plots of the four immune-related genes. The green line means enrichment profile.

Among the 13 immune-related hub genes, the expression of six genes showed significant differences between the MMD and the control groups. The results indicated that the expressions of *BTK, FGR, SYK, CSF2RB*, and *CXCR1* were upregulated in MMD, whereas the expression levels of PTPN11 were downregulated (Figure 10A). To verify the potential of immune-related hub genes as diagnostic biomarkers, we further downloaded the dataset GSE157628 as the external validation dataset. The expression pattern of *BTK, FGR, SYK*, and *PTPN11* showed consistent results (Figure 10B). The results indicated that *BTK, FGR, SYK*, and *PTPN11* genes showed strong stability and that they have the potential to be new diagnostic biomarkers. The correlation/anti-correlation among the 13 differentially expressed genes identified in the discovery cohorts was explored (Figure 10C). A positive correlation was demonstrated between *CSF2RB* and *CCR1*(r = 0.72) and *CXCR1* and *CCR1*(r = 0.74), whereas *TGFB2* has a negative correlation with *FGR* (r = −0.48). Moreover, GSEA was used to further explore the pre-defined immune-associated pathways of the four immune biomarkers. Single-gene GSEA results showed that several kinds of immune cell pathways were mainly enriched in MMD, including NK cells, monocyte cells, DC cells, and neutrophils (Figure 11).

**FIGURE 12:**
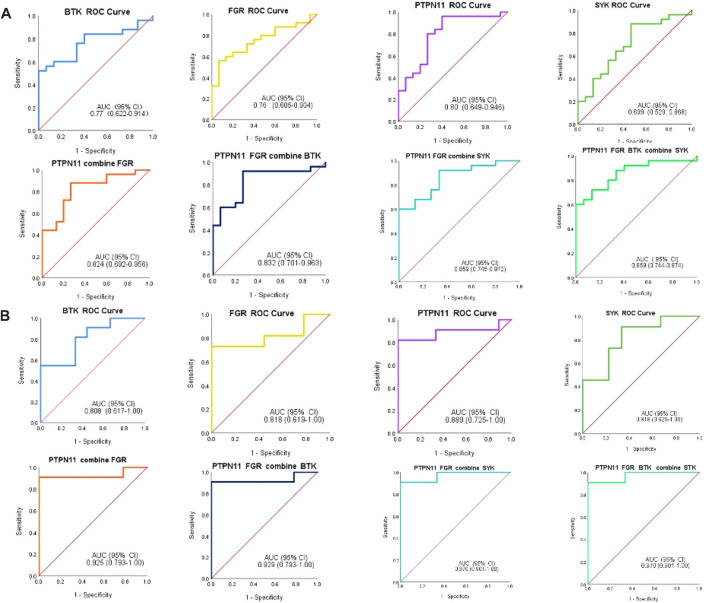
ROC analysis of immune-related biomarkers for diagnosis of MMD. **(A)** Single and combined ROC curves of four genes, namely, BTK, FGR, PTPN11, and SYK. Expression values of BTK, FGR, PTPN11, and SYK were extracted from the original expression matrix in samples of GSE189993 and GSE141022. **(B)** Single and combined ROC curves of the same four genes, and expression values of the same genes were extracted from microarray data in samples of GSE157628. The vertical axis means the specificity, and the horizontal axis means sensitivity.

In addition, we constructed the diagnostic model by using the logistic regression algorithm. ROC analysis was performed, and the four immune-related genes were visualized by ROC curves. ROC analysis confirmed that *BTK, FGR, SYK*, and *PTPN11* could distinguish MMD from control patients, and the accuracy of AUC ranged from 0.699 to 0.859 (Figure 12A). For single genes, *PTPN11* had the highest accuracy, with an AUC of 0.80. The results also revealed that the diagnostic model constructed by combined genes had better diagnostic performance, and the highest AUC was 0.859. Furthermore, in the validation set, the diagnostic power was much higher both in single and combined gene diagnostic models, with an AUC between 0.808 and 0.970 (Figure 12B). The results of the ROC analysis showed strong stability of the diagnostic performance between the different datasets, and *PTPN11* exhibited an excellent diagnostic capability. These identified four immune-related genes might function as novel diagnostic biomarkers and potential immunotherapeutic targets.

### Analysis of infiltrating immune cells

**FIGURE 13:**
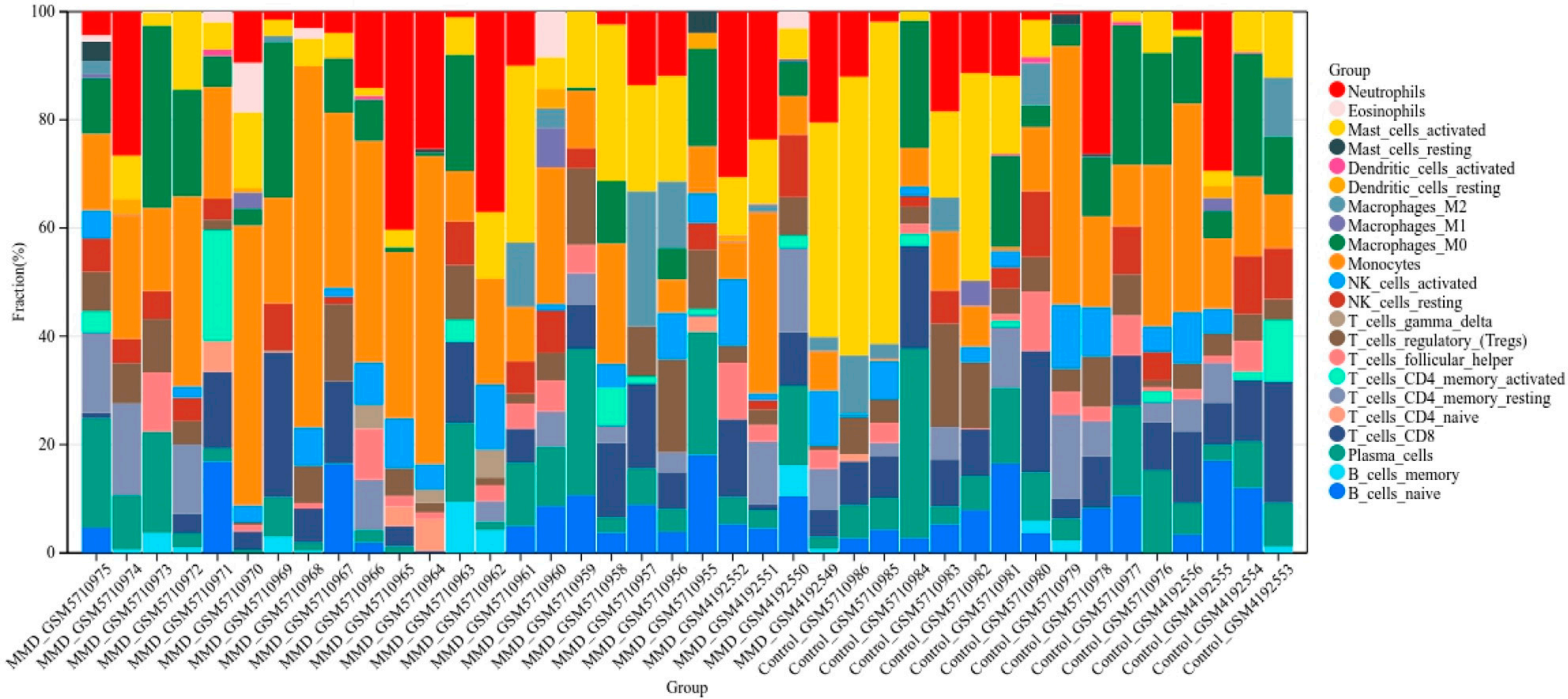
Bar charts of 22 immune cell proportions. Bar graph shows the percentage of immune cell subtypes between the two groups, where each color indicates each immune cell population.

**FIGURE 14:**
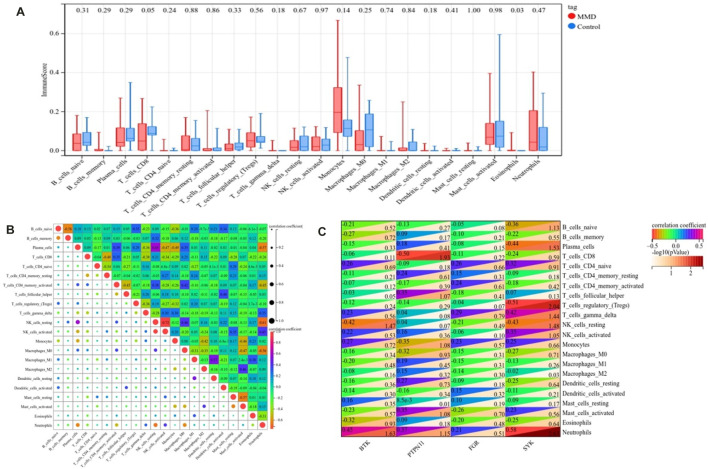
Landscape of immune infiltration. **(A)** Differential expression of immune cells between the MMD and control groups. **(B)** Correlation matrix of 22 immune infiltrating cell subtypes. **(C)** Correlation between intersecting core genes and 22 immune infiltrating cell subtypes. The vertical axis represents the immune cell subtypes, and the horizontal axis represents the core genes.

According to the CIBERSORT algorithm, the state of immune cell infiltration was reconstructed in MMD and the control group. Then, the immune infiltration score of 22 immune cell subtypes was obtained, and the proportions of immune cells in each sample are illustrated in Figure 13. The immune score results indicated that the fractions for eosinophils in the MMD group were remarkably higher than those of the control (*p* = 0.03). Compared with the control group, monocytes and neutrophils were upregulated in the MMD group, whereas CD8^+^ T cells, resting memory CD4^+^ T cells, and M0 macrophages were downregulated; however, they were not significant (Figure 14A). In addition, the correlation among the differentially infiltrated types of immune cells was analyzed, and several pairs of immune cell subtypes were found to have different degrees of positive or negative correlation (Figure 14B). This result suggests that M1 macrophages and resting DCs, M2 macrophages and activated mast cells, and M0 macrophages and resting NK cells showed the most synergistic effect. Together, resting NK cells and neutrophils, resting NK cells and native NK cells, and M0 macrophages and neutrophils represented the most competitive effect. Furthermore, the results of the in-depth relationship between the core genes and immune cell subtypes reported that *BTK* had a positive relationship with neutrophil infiltration and a negative relationship with resting NK cell infiltration, *PTPN11* had a positive relationship with neutrophil infiltration and a negative relationship with resting CD8 T cell infiltration, *FGR* showed a weak relationship with all the immune cell subtypes, and *SYK* had a positive relationship with neutrophil infiltration and a negative relationship with T regulatory cell (Tregs) infiltration (Figure 14C). These results fully indicated that the expression of immune-related genes had a strong correlation with immune infiltration.

## Discussion

Though MMD has been studied in the past 70 years, the underlying pathologies remain to be elucidated. The most prominent histopathologic characteristics of lumen narrowing or occlusion in MMD is the proliferation of smooth muscle cells and altered wavy, duplicated internal elastic lamina in intima, as well as progressive thinning of the tunica media (Suzuki and Kodama, 1983). In the past decades, autoimmune diseases have been observed to be co-occurrent with MMD, including autoimmune Graves’ disease, systemic lupus erythematosus, and type 1 diabetes (Watanabe et al., 2005; Kim et al., 2010; Bower et al., 2013). In addition, heavy deposition of immunoglobulins such as IgG and IgM was found in the thickening intima of vascular walls (Suzuki and Kodama, 1983), and an increased number of thyroid autoantibodies was reported in MMD patients, though there was no diagnosis of thyroid dysfunction (Kim et al., 2010). In addition, 165 significantly elevated autoantibodies were identified by the first high-throughput analysis of autoantibodies in the serum of MMD (Sigdel et al., 2013). Additionally, an integrated analysis of long non-coding RNA-messenger RNA co-expression networks reported their link to inflammatory response and the toll-like signaling pathway (Wang et al., 2017). Therefore, an increasing amount of evidence has showed that MMD is emerging as an immune-related angiopathy, triggered by immune-related responses as a second hit (Asselman et al., 2022). In our study, autoimmune thyroid disease and type-I diabetes mellitus were significantly screened according to GSEA-based enrichment, and they all suggested an underlying common pathogenic mechanism in these diseases. Antigen processing and presentation, primary immunodeficiency, and several other immune-related pathways were also enriched according to GO and KEGG enrichment of the identified hub genes. These results were consistent with the transcriptome-wide analysis results revealed by Kanamori F et al. of the upregulation of immune responses within the intracranial artery of MMD (Kanamori et al., 2021). The Fc epsilon RI signaling pathway and Jak-STAT signaling pathway are two significant pathways mainly involved in the progression of MMD. Emerging evidence reveals that inflammatory or immune activities may present as a second hit in the progression of MMD. Taken together, an increasing amount of evidence suggests a close relationship between the immunity reaction activities and development of MMD.

Subsequently, we screened the immune-related diagnostic biomarkers for MMD and validated them in the external dataset. As we know, biomarkers are biological fingerprints and could be used to clarify a specific disease. A panel of urinary biomarkers (Sesen et al., 2021), metabolic biomarkers (Geng et al., 2020), radiographic biomarkers (Storey et al., 2017), circulating endothelial progenitor cells and endothelial cells (Bao et al., 2018), miRNAs (Dai et al., 2014; Wang G. et al., 2020; Wang et al., 2021), and a series of potential molecules from blood or cerebral spinal fluid (Smith, 2015) have been reported previously. However, there is still no well-recognized biomarker for accurately diagnosing and predicting the outcome of MMD. *CD38, PTPN11, NOTCH1, TLR7, KAT2B,* and *ISG15* were identified using machine learning, and a reliable diagnostic model was developed by Li et al. (2022). Except *RNF213, UNC13D* was previously identified as a key biomarker with good specificity and sensitivity (Jin and Duan, 2022). In the present research, 13 immune genes were selected from DEGs and the most significant module genes. *BTK, FGR, PTPN11*, and *SYK* were identified as hub immune-associated genes. Due to the lack of a recognized cellular model for MMD, the cell functions of these genes were not verified in MMD research. However, validation was performed in external data, and the results showed strong predictive stability. They have the potential to be diagnostic biomarkers for MMD. Among them, PTPN11 showed a low expression pattern and the highest diagnostic accuracy, and it also showed the highest degree according to PPI network analysis, which was identified by Li et al. (2022), as aforementioned. *PTPN11* encodes a non-receptor tyrosine phosphatase with 2 Src-homology 2 domains (Shp2) as well, and it was identified as the first proto-oncogene. *PTPN11* is ubiquitously expressed in somatic cells and has important functions in regulating cell proliferation, differentiation, migration, and adhesion. Additionally, *PTPN11* could positively or negatively regulate the intracellular kinase-mediated signaling pathways (Tajan et al., 2015), for example, upregulate or downregulate the activities of PI3/AKT (Zhang et al., 2002) and JAK/Stat signaling (Ke et al., 2006). Consistently, the JAK/Stat signaling pathway was enriched by functional analysis. Especially, mutations in *PTPN11* have been reported in malignant hematological diseases and solid tumors (Bentires-Alj et al., 2004; Siegfried et al., 2017; Alfayez et al., 2021). Furthermore, the *PTPN11* gene causes Noonan syndrome, accounting for approximately 50% of cases of this genetic disorder (Tartaglia et al., 2002). Noonan syndrome is known to cause moyamoya syndrome (MMS); the association between the two diseases was described first in 1997 (Ganesan and Kirkham, 1997), and an increasing number of patients have been reported recently (Tang et al., 1999; Yamashita et al., 2004; Hung et al., 2011; Choi et al., 2015). However, the potential molecular mechanisms of *PTPN11* in MMD remain to be further explored.

Finally, we performed the analysis of 22 types of immune cell infiltrates in MMD tissue using CIBERSORT. In recent decades, the role of immune infiltrates has become increasingly evident. Although immune infiltration was not typical in the pathology of MMD arteries, Masuda *et al.* did report immune cell infiltrating components such as macrophages or T cells in the thickened vascular wall (Masuda et al., 1993). Recently, Li et al. reported the significant difference of immune infiltration in eosinophils, natural killer T (NKT) cells, and Th2 cells between MMD and controls (Li et al., 2022), whereas Jin and Duan (2022) demonstrated an elevated abundance of neutrophils, monocytes, and natural killer cells in MMD. However, the detailed mechanism of immune cell function in MMD was still largely unknown. In the present study, the infiltrated immune cell populations of the MCA vascular wall were compared, and the results showed a significant difference for the presence of eosinophils, consistent with Li et al’s findings. Eosinophils are white blood cells, and they take part in a series of biologic processes. Eosinophils and their secretory products such as granule proteins and chemical mediators are important in maintaining physiological homeostasis in both normal physiologic homeostasis and disease pathology (Wechsler et al., 2021). Their role in the cardiocirculatory and the nervous system, respiration and immune function, and tissue metabolism and remodeling has been described recently (Idzko et al., 2014; Burnstock, 2017; Burnstock and Gentile, 2018). Infiltrated eosinophils also respond to hypoxia by increasing their viability and proangiogenic potential (Gonlugur and Efeoglu, 2004). Due to their important role in innate immunity and modulation of angiogenesis, an alternative treatment strategy targeting eosinophils could represent a novel approach. Our comparison of immune infiltration also showed that the MMD group contained a higher number of monocytes and neutrophils, in contrast to a lower level of CD8^+^ T cells, resting memory CD4^+^ T cells, and M0 macrophages. However, there was no significance between both the number of increased and decreased immune infiltrating cells. According to GSEA-based MSigDB enrichment, several immunocyte subtypes were detected in the pathways, including monocytes, neutrophils, DCs, NK cells, and macrophages. The results were in line with those of immune infiltration. In addition, the relationship between immune infiltration and the immune-related core genes has been further explored. As *PTPN11* has the highest diagnostic accuracy, it was found that *PTPN11* was positively correlated with neutrophil infiltration but negatively with the CD8 T cell.

Based on these findings of immune-related genes, immune pathways, and immune infiltration, this research has offered a new viewpoint on the immune infiltration of MMD. Therefore, it is helpful for understanding the potential pathological mechanisms of MMD and immunotherapy targets. Recently, bioinformatics analysis has been found to be a functional tool in understanding human diseases, and the potential effects of infiltrating immune cells on MMD arteries should be investigated with modern bioinformatics tools or even single-cell technologies. Despite the great significance, there were some limitations in the study. First, the research had a relatively small sample size, though it was the biggest microarray data update. Next, we lacked further vitro cytology experiments and verification of cell functions. Owing to the complex functions of gene molecules, these possible variations of bioinformatics results need to be further verified.

## Conclusion

Our research provided a new viewpoint of the immune landscape in the immune mechanism of MMD development. Eosinophils revealed the greatest differences in the analysis of immune infiltration. Immune response was mainly associated with the pathogenesis of MMD. Four immune-related hub genes (*PTPN11, BTK, FGR,* and *SYK*) were identified and further analyzed as candidate biomarkers for MMD. These genes and immune cells may perform crucial functions and were identified as novel targets for immunotherapy.

## Data Availability

The datasets analyzed in this study were downloaded and accessed from the Gene Expression Omnibus (GEO) database: https://www.ncbi.nlm.nih.gov/geo/, with accession nos. GSE189993, GSE141022, and GSE157628.

## Abbreviations

AUC, area under the curve; BP, biological process; CC, cellular component; CIBERSORT, cell-type identification by estimating relative subsets of RNA transcript; DEGs, differentially expressed genes; GEO, Gene Expression Omnibus; GO, Gene Ontology; GSEA, gene set enrichment analysis; KEGG, Kyoto Encyclopedia of Genes and Genomes; MF, molecular function; MSigDB, Molecular Signature Database; PPI, protein–protein interaction; WGCNA, weighted gene co-expression network analysis.
